# The IGF system in patients with inflammatory bowel disease treated with prednisolone or infliximab: potential role of the stanniocalcin-2 / PAPP-A / IGFBP-4 axis

**DOI:** 10.1186/s12876-019-1000-6

**Published:** 2019-06-03

**Authors:** Rikke Hjortebjerg, Karen L. Thomsen, Jørgen Agnholt, Jan Frystyk

**Affiliations:** 1 Medical Research Laboratory, Department of Clinical Medicine, Faculty of Health, Aarhus University, Aarhus, Denmark; 2 The Danish Diabetes Academy, Odense, Denmark; 3 Department of Hepatology and Gastroenterology, Aarhus University Hospital, Aarhus, Denmark; 4 Department of Clinical Research, Faculty of Health, University of Southern Denmark, Odense, Denmark; 5 Department of Endocrinology, Odense University Hospital, Odense, Denmark

**Keywords:** IGF binding protein-4, Inflammatory bowel disease, Infliximab, Prednisolone, Pregnancy-associated plasma protein-a, Stanniocalcin-2

## Abstract

**Background:**

Patients with inflammatory bowel disease (IBD) present with reduced serum insulin-like growth factor I (IGF-I). Anti-inflammatory treatment with prednisolone or infliximab ameliorates symptoms and increases circulating IGF-I, but prednisolone induces catabolism, whereas infliximab may promote protein synthesis. Recently, stanniocalcin-2 (STC2) was discovered as a novel inhibitor of the enzyme pregnancy-associated plasma protein-A (PAPP-A), which modulates IGF-I activity. PAPP-A can cleave IGF binding protein-4 (IGFBP-4), upon which IGF-I is liberated. We hypothesized that prednisolone and infliximab exert different effects on levels of STC2, PAPP-A, and IGFBP-4, thereby explaining the distinct metabolic effects of prednisolone and infliximab.

**Methods:**

Thirty-eight patients with active IBD treated with either prednisolone (*n* = 17) or infliximab (*n* = 21) were examined before and after 7 days of treatment. Circulating levels of IGF-I, IGF-II, IGFBP-3, PAPP-A, and STC2 were measured by immunoassays. Intact IGFBP-4 and two IGFBP-4 fragments were determined by a novel immunoassay. Bioactive IGF was assessed by cell-based IGF receptor activation assay. Concentrations of IGFBP-4, PAPP-A, and STC2 on day 0 and 7 were compared to healthy control subjects.

**Results:**

Following seven days of prednisolone treatment, total and bioactive IGF-I were increased (*p* < 0.001 and *p* < 0.05, respectively). Upon infliximab treatment, total IGF-I levels were augmented (p < 0.05), yet IGF bioactivity remained unaltered. Intact IGFBP-4 and the two IGFBP-4 fragments generated upon cleavage by PAPP-A were all decreased following treatment with either prednisolone or infliximab (all *p* < 0.05). PAPP-A levels were only increased by infliximab (*p* = 0.005), whereas the inhibitor STC2 did not respond to any of the treatments.

**Conclusion:**

IGF-I and IGFBP-4 concentrations were markedly altered in patients with IBD and near-normalized with disease remission following treatment with prednisolone or infliximab. Thus, IGFBP-4 may modulate IGF bioavailability in IBD. The effect of immunosuppression did not appear to extend beyond the regulation of IGF and IGFBP-4, as neither PAPP-A nor STC2 were discernibly affected.

**Trial registration:**

ClinicalTrials.gov: NCT00955123. Date of registration: August 7, 2009 (retrospectively registered).

## Background

Inflammatory bowel diseases (IBD), such as Crohn’s disease and ulcerative colitis, are chronic gastrointestinal disorders with unknown etiology [1, 2]. The genetically predisposed IBD patients are characterized by an abnormal intestinal mucosal immune response against the commensal intestinal flora, but the pathogenesis remains poorly understood. Patients present with an ongoing systemic auto-inflammation and various metabolic deteriorations, including submucosal fat deposition, insulin and growth hormone (GH) resistance, hypertension, and dyslipidemia [2, 3]. Current treatment strategies in IBD include corticosteroids and anti-inflammatory biological agents. Prednisolone is a mainstay in the treatment of active IBD, despite numerous deleterious catabolic side-effects such as accelerated protein wasting, decreased protein synthesis, and bone demineralization. In the past two decades, the use of anti-inflammatory biological agents, including antibodies against tumor necrosis factor-α (TNF-α) (infliximab), has become ubiquitous, both as a first-line treatment and when treatment with prednisolone fails to control the inflammation [1].

Patients with active IBD present with reduced serum levels of insulin-like growth factor I (IGF-I) [4, 5], likely secondary to a combination of GH resistance, gastrointestinal dysfunction, and chronic inflammation [3, 6, 7]. IGF-I governs anabolic cellular processes as well as carbohydrate, lipid and protein metabolism [8, 9]. The biological activity of IGF-I is modulated by a family of IGF binding proteins (IGFBPs) as well as a number of IGFBP proteases. Its cognate receptor, the IGF-I receptor (IGF-IR), is ubiquitously expressed in most tissue, including the gastrointestinal tract [6]. Reduction in systemic inflammation following various therapeutic interventions partly reverses GH resistance and often results in near-normalization of circulating IGF-I levels [3, 7]. Interestingly, exposure to prednisolone generally suppresses IGF-I bioactivity within the tissues, while increasing serum IGF-I concentration and ability to activate the IGF-IR in vitro (bioactive IGF) [10]. The paradox appears unrelated to changes in the circulating levels of the IGFBPs, whereas previous studies have suggested that IGF bioactivity may be regulated within the tissues by unknown IGF-I inhibitors [11–13].

**Fig. 1:**
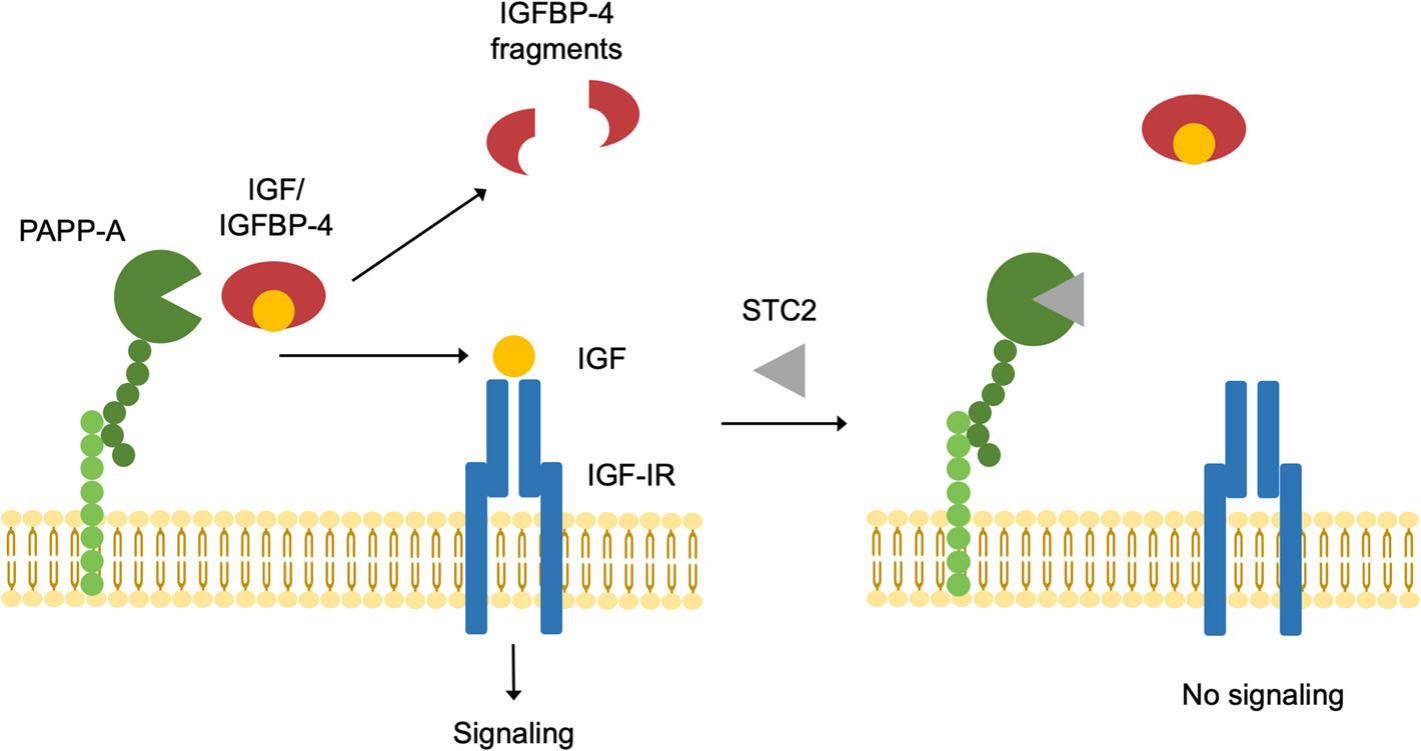
The stanniocalcin-2/PAPP-A/IGFBP-4 axis. Left: PAPP-A cleaves IGFBP-4 into two fragments in the vicinity of the IGF-IR, resulting in liberation of bioactive IGF that can activate its receptor. Right: stanniocalcin-2 inhibits PAPP-A degradation of IGFBP-4, resulting in decreased levels of bioactive IGF and consequently reduced IGF signaling. IGF, insulin-like growth factor; IGF-IR, IGF-I receptor; IGFBP, IGF binding protein; PAPP-A, pregnancy-associated plasma protein-A; STC2, Stanniocalcin-2

Pregnancy-associated plasma protein-A (PAPP-A) is an enzyme with proteolytic activity towards IGFBP-4 [14, 15]. However, IGFBP-4 is only subject to degradation when bound to the IGFs, and as proteolysis renders IGFBP-4 unable to bind its ligand, PAPP-A hereby serves as a stimulator of IGF-I action by liberating IGF [14, 16]. Recently, stanniocalcin-2 (STC2) was identified as a novel inhibitor of PAPP-A [17]. Binding of STC2 to PAPP-A abrogates the enzymatic activity towards IGFBP-4, hereby indirectly decreasing IGF bioactivity (Fig. 1). The effects of the STC2/PAPP-A/IGFBP-4-axis [18] on IGF activity has been linked to a number of pathological conditions, including adipose tissue dysfunction [19, 20], cardiovascular disease [14, 21–24], and cancer [16, 25]. Additionally, PAPP-A is notoriously stimulated by pro-inflammatory cytokines, and thus, we hypothesized that IBD treatments, via their anti-inflammatory effects, may impinge on IGF-I activity via the STC2/PAPP-A/IGFBP-4-axis [26]. Accordingly, the present study aimed to compare the effects of treatment with prednisolone vs. infliximab on the recently acknowledged STC2/PAPP-A/IGFBP-4-axis in patients with active IBD.

## Methods

### Patients and study design

Details of the study participants and procedures have previously been described [27]. A total of 38 consecutive patients with active IBD were enrolled, of which 20 patients presented with ulcerative colitis and 18 with Crohn’s disease. The patients had been diagnosed according to the standard clinical, biochemical, endoscopic, and histopathological criteria. Inclusion criteria were age above 18 years and moderate-to-severe active ulcerative colitis or Crohn’s disease that required systemic anti-inflammatory treatment with prednisolone or infliximab. Thus, all patients exhibited clinically active, inflammatory disease on the basis of standard criteria, including elevated C-reactive protein (CRP), erythrocyte sedimentation rate (ESR), orosomucoid, and fecal calprotectin, and disease activity was furthermore scored according to the Harvey Bradshaw Index (HBI) [28] in patients with Crohn’s disease and the Simple Clinical Colitis Activity Index (SCCAI) [29] in patients with ulcerative colitis. All patients were bio-naïve, but had previously been treated with azathioprine (*n* = 8), 5-aminosalicylic acid (*n* = 13), both azathioprine and 5-aminosalicylic acid (*n* = 2) or budesonide (n = 2). Patients treated with budesonide were not assigned to the prednisolone treatment group. Thirteen patients were newly diagnosed or treatment naïve. Exclusion criteria were the use of either prednisolone or infliximab within 8 weeks prior to enrolment. Furthermore, patients with active infections, malignancies, or other catabolic or chronic inflammatory diseases were excluded. Of the included patients, 17 were treated with prednisolone (13 ulcerative colitis and 4 Crohn’s disease patients), and 21 were treated with infliximab (7 ulcerative colitis and 14 Crohn’s disease patients). The department’s gastroenterologists made the treatment decision according to clinical criteria and before informed consent was obtained from the participant. Intravenous treatment was either with methylprednisolone (40 mg twice daily) (Solu-Medrol, Pfizer, NY, USA) or infliximab (5 mg/kg body weight, one dose) (Remicade; Janssen Biologics B.V., Leiden, Netherlands). Patients receiving infliximab treatment had previously experienced steroid resistance or had severe adverse effects in response to steroid treatment. The two treatment groups were balanced with regard to age, sex, height, and weight. All investigations were performed after an overnight fast. Blood samples were collected from all patients at baseline (day 0) and on day 7 and stored at − 80 °C until analysis.

To specifically characterize the STC2/PAPP-A/IGFBP-4-axis in IBD patients, protein measurements on day 0 and day 7 were compared to healthy control subjects. Control EDTA-plasma for IGFBP-4 and STC2 measurements was collected from 50 randomly selected Danish registered blood donors at Aarhus University Hospital (25 women and 25 men, age range 18–50 years). Control serum for PAPP-A measurements was collected from 150 adults (75 women and 75 men, age 44 ± 15 years) [30]. All donors were healthy and received no medication.

The study was approved by The Local Ethics Committee (Journal no. 20060197) and registered at ClinicalTrials.gov (NCT00955123). Written informed consent was obtained from all participants, and the study conformed to the Declaration of Helsinki.

### Laboratory measurements

CRP, ESR, orosomucoid, and creatinine were assayed using routine laboratory methods. Estimated glomerular filtration rate (eGFR) was calculated using the MDRD formula [31]. Cortisol and fecal calprotectin were measured by specific ELISAs (DRG Diagnostics, Marburg, Germany and Bühlmann Laboratories, Schönenbuch, Switzerland, respectively), and TNF-α and IL-6 by highly sensitive immunoassays (RnD Systems, Minneapolis, MN, USA).

Total serum IGF-I was assayed after acid-ethanol extraction using a validated in-house sandwich assay as previously described [32] with minor modifications: the secondary detection antibody was replaced by a biotinylated IGF-I antibody (Cat# I-8773, Sigma-Aldrich, St. Louis, MO, USA). Serum IGF-II concentrations were measured by a validated in-house, time-resolved immunofluorometric assay (TR-IFMA) [33]. IGFBP-3 was determined using the IDS-iSYS Multi-Discipline Automated Analyzer (Immunodiagnostic Systems, Copenhagen, Denmark), as previously published [22].

The ability of serum IGFs to activate the IGF-IR in vitro (bioactive IGF) was determined by an in-house kinase receptor activation assay (KIRA) as originally described [34] with slight modifications. The assay measures the ability of IGF to phosphorylate the IGF-IR in an in vitro-based model employing IGF-IR gene-transfected human embryonic renal cells. In brief, transfected cells were stimulated with diluted serum for 15 min at 37 °C. A serial dilution of rhIGF-I (WHO 02/254) served as calibrator. Following cell lysis, the concentration of phosphorylated IGF-I receptor was determined in the crude cell lysates using a phospho-IGF-IR ELISA (Cat# DYC 1770E, RnD Systems, Abingdon, UK). The KIRA assay signal primarily reflects binding of IGF-I to the IGF-IR, but also binding of IGF-II and pro-IGF-II (cross-reactivity 12 and 2%, respectively). The cross-reactivity of pro-insulin and insulin is negligible (< 1%). Hence, the assay signal is referred to as IGF bioactivity. The KIRA assay has a detection limit < 0.08 μg/L and intra- and inter-assay CVs of 12 and 20%, respectively.

EDTA-plasma levels of intact IGFBP-4 and the two PAPP-A generated fragments, C-terminal (CT)-IGFBP-4 and N-terminal (NT)-IGFBP-4, were measured in duplicate by in-house TR-IFMAs using monoclonal antibodies (mAb) and recombinant human (rh) calibrators generously provided by HyTest Ltd. (Turku, Finland). The assays were performed as recently described [14, 21–23]. In each fragment assay, one of the antibodies specifically recognized the proteolytic neoepitope generated upon cleavage by PAPP-A. Detection limits were 0.5 μg/L for IGFBP-4, 0.4 μg/L for CT-IGFBP-4 and 0.9 μg/L for NT-IGFBP-4. Intra- and inter-assay CVs were < 10 and < 15%, respectively.

PAPP-A and STC2 levels were determined by commercial sandwich ELISAs (PAPP-A; Cat# AL-101 and STC2; Cat# AL-143) from AnshLabs (Webster, TX, USA). Assay procedures were as described by the manufacturer, and both assays behaved linearly within the analytical range.

### Statistics

Baseline data have previously been described and analyzed [27]. The assumption of normality was checked using quantile plots, and non-normally distributed variables were transformed using the natural logarithm prior to statistical analyses. Unpaired continuous observations were analyzed at baseline using Student’s t-test, and paired observations on day 0 and day 7 were analyzed using paired t-test. Variables that remained non-normally distributed upon transformation were analyzed using the nonparametric Wilcoxon matched-pairs signed-ranks test or Mann Whitney *U*-statistics as appropriate. Categorical variables were compared using χ^2^-test. Differences in protein concentrations between IBD patients and controls were assessed by unpaired t-test. Correlations were examined using Pearson’s correlation analysis. To investigate the overall change from day 0 to day 7 in IGF system parameters and inflammatory and metabolic markers, we calculated delta values in each subject. Correlations between delta values were investigated using Pearson or Spearman correlation coefficient as appropriate. The proteins investigated in the present study have previously shown to provide ample statistical power in cohorts of 9–24 subjects [10, 35, 36], supporting that differences in proteins levels would be detectable in a cohort of 38 patients. Data are reported as means ± SD for normally distributed variables and medians with interquartile range for non-normally distributed variables. Level of significance was *p* < 0.05. Statistical analyses were performed using STATA 13 (StataCorp, College Station, TX, USA).

## Results

### Baseline characteristics and disease activity

Measurement details of healthy subjects and IBD patients before and after treatment are summarized in Table 1*.* At baseline, disease scores, CRP, ESR, orosomucoid, fecal calprotectin, cortisol, TNF-α and IL-6 were higher compared with levels in healthy subjects (reference levels not shown), but similar in the two treatment groups. The eGFR was slightly higher in the infliximab group. Irrespective of anti-inflammatory treatment, patient SCCAI and HBI disease scores as well as CRP, ESR, orosomucoid, and fecal calprotectin improved during the course. Both treatments resulted in reduced IL-6 levels, whereas infliximab triggered a possibly compensatory increment in TNF-α levels. These data have previously been published [27].

**Table 1:** Baseline characteristics and protein levels at baseline and 7 days after treatment with prednisolone or infliximab. Data are mean ± SD or median (25th percentile; 75th percentile)

Characteristics	Healthy subjects	Prednisolone (*n* = 17)		Infliximab (*n* = 21)		
		Day 0	Day 7	Day 0	Day 7	*P*_*Group, day 0*_
Ulcerative colitis/ Crohn’s disease (n)		13/4		7/14		0.008
Age (years)		34.4 ± 8.9		30.5 ± 11.0		0.243
Male/female (n)		10/7		10/11		0.492
BMI (kg/m2)		23.3 ± 4.5		25.1 ± 5.8		0.306
SCCAI		9 (7;10)	2 (1;2)**	8 (5;9)	2 (1;4)*	0.537
HBI		8 (5;13)	4 (1;6)*	5.5 (3;8)	2 (1;3)**	0.243
eGFR		98 (70;104)	94 (89;101)	102 (94;122)	101 (93;114)	0.008
CRP (mg/L)		12.8 (3.6;21.5)	7.2 (3.5;21.2)**	7.2 (3.5;21.2)	2.7 (1.4;7.9)*	0.587
ESR (mm/h)		14 (7;27)	6 (5;10)**	13.5 (8.5;19.5)	11 (7;16)*	0.647
Orosomucoid (g/L)		1.4 ± 0.4	1.2 ± 0.5*	1.3 ± 0.4	1.0 ± 0.3**	0.383
Calprotectin (mg/kg)		2072 ± 1254	1100 ± 1124**	1569 ± 1099	810 ± 777**	0.311
Cortisol (μg/L)		129 (107;255)	435 (27;941)	125 (104;154)	129 (110;141)	0.364
Insulin (pmol/L)		44 ± 38	42 ± 17	45 ± 25	49 ± 26	0.865
TNF-α (ng/L)		1.5 ± 1.0	1.1 ± 1.9	1.1 ± 0.9	11.5 ± 6.0**	0.173
IL-6 (ng/L)		2.9 ± 2.6	1.6 ± 1.5*	2.8 ± 1.6	1.7 ± 1.7*	0.973
IGF-I (μg/L)		128 ± 36	185 ± 67**	159 ± 33	178 ± 50*	0.009
IGF-II (μg/L)		502 ± 61	502 ± 60	495 ± 71	518 ± 75*	0.746
IGF bioactivity (μg/L)		1.08 ± 0.36	1.34 ± 0.52*	1.23 ± 0.42	1.33 ± 0.45	0.260
IGFBP-3 (μg/L)		4120 ± 583	4332 ± 771	4220 ± 687	4417 ± 687*	0.638
IGFBP-4 (μg/L)	174 ± 79	184 (146;315)	132 (111;188)*	183 (152;207)	164 (97;209)*	0.410
CT-IGFBP-4 (μg/L)	72 ± 18	112 ± 33†	79 ± 41**	99 ± 35†	78 ± 35**	0.246
CT-IGFBP-4/IGFBP-4 ratio	0.41 (0.29;0.53)	0.61 ± 0.27†	0.57 ± 0.35†	0.55 ± 0.15†	0.59 ± 0.36†	0.443
NT-IGFBP-4 (μg/L)	153 ± 41	257 ± 111†	179 ± 93**	207 ± 76†	170 ± 78**	0.108
NT-IGFBP-4/IGFBP-4 ratio	0.80 (0.61;0.99)	1.34 ± 0.57†	1.28 ± 0.72†	1.16 ± 0.31†	1.30 ± 0.84†	0.223
PAPP-A (μg/L)	0.81 ± 0.26	1.17 ± 0.66†	1.21 ± 0.56†	0.76 ± 0.37	0.87 ± 0.33*	0.019
STC2 (μg/L)	25.0 (21.2;28.1)	22.2 ± 5.2	21.5 ± 4.8	22.9 ± 5.6	24.3 ± 7.6	0.698

Categorical variables are indicated as numbers (n) of patients. Comparison of groups on day 0 was performed by Student’s t-test, and comparison of observations on day 0 and day 7 within the same treatment group were performed using paired t-test. CRP, ESR, and cortisol were analyzed using the nonparametric Wilcoxon matched-pairs signed-ranks test or the Mann–Whitney *U*-statistics as appropriate. Differences in protein concentrations between patients and controls were assessed by unpaired t-test. Categorical variables were compared using χ^2^-test. *P < 0.05, **P < 0.005 when compared to day 0 within the same treatment group. †*p* < 0.05 for differences between IBD patients and controls on day 0 or day 7

*BMI* body mass index, *CRP* C-reactive protein, *CT* C-terminal, *eGFR* estimated glomerular filtration rate, *ESR* erythrocyte sedimentation rate, *HBI* Harvey–Bradshaw Index, *IGF* insulin-like growth factor, *IGFBP* IGF binding protein, *IL-6* interleukin-6, *NT* N-terminal, *PAPP-A* pregnancy-associated plasma protein-A, *SCCAI* Simple Clinical Colitis Activity Index, *STC2* Stanniocalcin-2, *TNF-α* tumour necrosis factor-α

### The IGF system in patients with IBD treated with prednisolone or infliximab

**Fig. 2:**
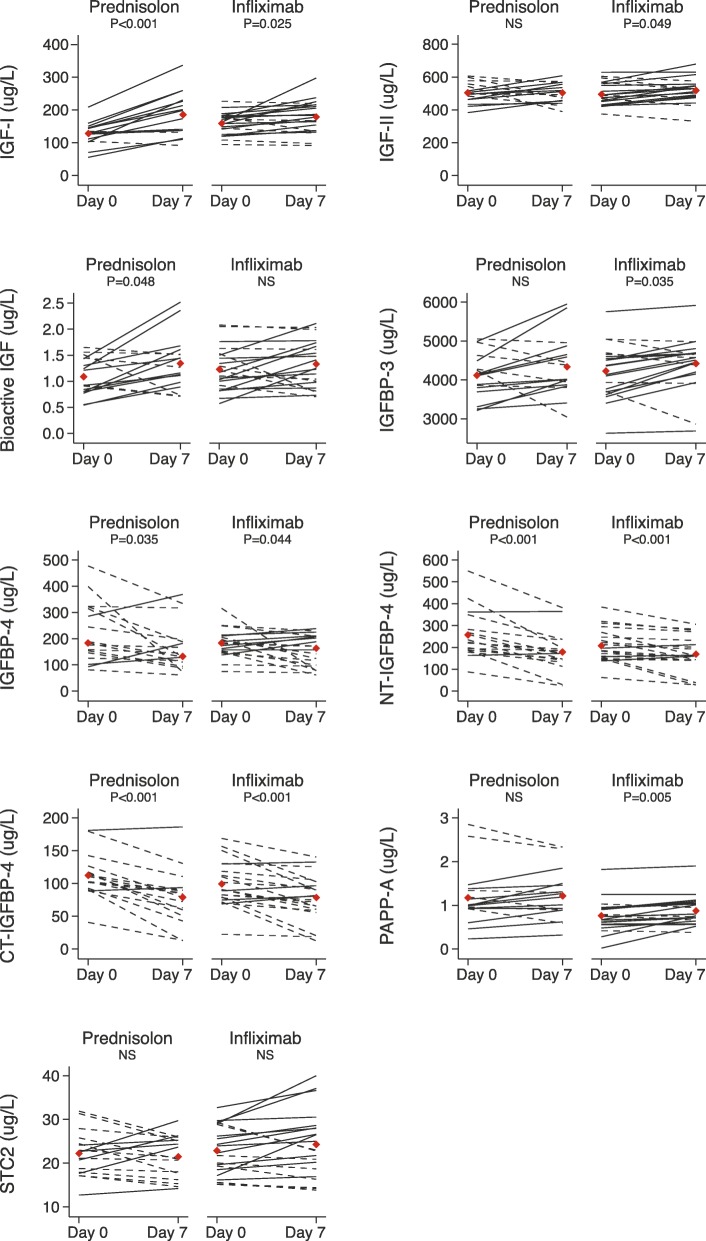
Line plots illustrating individual changes in IGF system protein levels from day 0 to day 7 after treatment with prednisolone or infliximab. The individual changes in total IGF-I, IGF-II, bioactive IGF, IGFBP-3, IGFBP-4, NT-IGFBP-4, CT-IGFBP-4, PAPP-A, and STC2 before and after 7 days of prednisolone or infliximab. Solid lines illustrate patients, in which protein levels were increased following therapy, whereas dashed lines show patients that had lowered protein levels. Red diamonds illustrate mean or median protein level before and after treatment. CT, C-terminal; IGF, insulin-like growth factor; IGFBP, IGF binding protein; NS, not significant; NT, N-terminal; PAPP-A, pregnancy-associated plasma protein-A; STC2, Stanniocalcin-2

Paired individual levels of IGF system proteins are shown in Fig. 2. At baseline, IGF-I levels were higher in the infliximab group, whereas IGF-II, IGF bioactivity, and IGFBP-3 levels were similar in the two treatment groups (Table 1). Following seven days of prednisolone treatment, both total IGF-I concentration and bioactive IGF, as measured by in vitro IGF-IR activation, were significantly increased (*p* < 0.001 and *p* = 0.048, respectively). In contrast, concentrations of IGF-II and IGFBP-3 remained unchanged. When looking at the individual changes, six patients demonstrated numerically lower levels of bioactive IGF after prednisolone treatment, whereas 11 patients showed numerically higher levels following prednisolone. By contrast, the concentration of IGF-I was only reduced in two patients. Upon infliximab treatment, levels of IGF-I, IGF-II, and IGFBP-3 were slightly increased (*p* = 0.025, *p* = 0.049, and *p* = 0.035, respectively), whereas IGF bioactivity remained unaltered.

### The STC2/PAPP-A/IGFBP-4-axis

#### Patients with IBD vs. healthy control subjects

Neither on day 0 nor day 7 did intact IGFBP-4 levels differ from those of healthy controls. However, CT- and NT-IGFBP-4 levels were significantly higher at baseline in both the prednisolone and infliximab group (all *p* < 0.001), and both protein concentrations were normalized upon treatment. To assess the degree of IGFBP-4 degradation, we calculated ratios between each fragment and intact IGFBP-4. As compared to healthy controls, the CT-IGFBP-4/IGFBP-4 and NT-IGFBP-4/IGFBP-4 ratios were significantly higher on day 0 and 7 in both the prednisolone and infliximab group (all *p* < 0.005), suggesting an increased proteolytic degradation that was not remedied by treatment. In the prednisolone group, PAPP-A levels were above normal both before and after therapy (all *p* < 0.001). STC2 levels were similar in IBD patients and controls.

#### Patients with IBD treated with prednisolone or infliximab

Prior to treatment, IGFBP-4, IGFBP-4 fragments, and STC2 concentrations were of similar magnitude in the two groups, whereas PAPP-A levels were higher in the prednisolone group. Intact IGFBP-4 was reduced upon both prednisolone as well as infliximab treatment (*p* = 0.035 and *p* = 0.044, respectively), and similar responses were observed for CT-IGFBP-4 and NT-IGFBP-4 (all *p* < 0.001). Since both intact and fragmented IGFBP-4 decreased in parallel, ratios between intact IGFBP-4 and each fragment were inspected at each time point. Independent of treatment, the CT-IGFBP-4/IGFBP-4 and NT-IGFBP-4/IGFBP-4 ratios remained unaltered during the course, and thus, the relative degradation of IGFBP-4 on day 0 and day 7 appeared to be of similar magnitude. PAPP-A levels remained unaffected by prednisolone but were significantly increased by infliximab (*p* = 0.005). The PAPP-A inhibitor STC2 did not respond to any of the treatments.

### Associations between IGF system proteins and inflammatory and metabolic parameters

Total IGF-I and IGF bioactivity were not correlated on day 0 (r = 0.22, *p* = 0.19), but were strongly associated on day 7 (r = 0.72, *p* < 0.001). At baseline, intact IGFBP-4 was negatively correlated with PAPP-A (r = − 0.31, *p* < 0.05) and positively correlated with CT- and NT-IGFBP-4 (r = 0.45, *p* < 0.005 and r = 0.48, p < 0.005, respectively). In addition, a strong positive correlation was observed between concentrations of CT- and NT-IGFBP-4 at both time points (day 0: r = 0.84, *p* < 0.001, and day 7: r = 0.93, *p* < 0.001). CT- and NT-IGFBP-4 were positively associated with TNF-α at baseline (r = 0.34, *p* < 0.05 and r = 0.44, *p* < 0.01, respectively), and negatively associated with eGFR (day 0: r = − 0.48, *p* < 0.005 and r = − 0.62, *p* < 0.001, respectively, and day 7: r = − 0.34, *p* < 0.05 and r = − 0.43, p < 0.01, respectively). IGF system proteins did not correlate with CRP, IL-6 or the functional hepatic nitrogen clearance at either time point.

To investigate the individual change from baseline in IGF system parameters within each subject, we calculated the delta values from day 0 to day 7. We observed a positive association between the delta value of IGF-I and IGF bioactivity from day 0 to day 7 (r = 0.47, *p* < 0.005). Correlations were also observed between the change in CT- and NT-IGFBP-4 and the change in PAPP-A (r = 0.32, *p* < 0.05 and r = 0.37, p < 0.05, respectively). Thus, the patients that experienced the most substantial increase in fragmented IGFBP-4 also demonstrated the largest increase in PAPP-A. The shift in intact IGFBP-4 from day 0 to day 7 was positively associated with the change in STC2 (r = 0.48, p < 0.005). Accordingly, patients with the most massive reductions in intact IGFBP-4 also showed the most pronounced declines in levels of the PAPP-A inhibitor STC2.

## Discussion

Prednisolone and infliximab represent two distinct anti-inflammatory treatments of patients with IBD. Both treatments effectively alleviate active disease, albeit acting through different mechanisms and having distinct metabolic effects. Prednisolone induces catabolism, whereas infliximab may promote protein synthesis [27]. Still, both treatments increase serum IGF-I concentrations [3, 5, 7, 37]. We hypothesized that prednisolone and infliximab would have distinct effects on a recently acknowledged regulatory system that controls IGF-I action independently of circulating IGF-I concentrations; the STC2/ PAPP-A/ IGFBP-4-axis. This axis has been associated with numerous inflammatory states [16, 25], and thus, anti-inflammatory treatment may likely influence on the STC2/PAPP-A/IGFBP-4-axis [26]. To this end we compared the responses of the IGF system in IBD patients treated for 7 days with prednisolone or infliximab, paying specific attention to STC2, PAPP-A, and IGFBP-4. In agreement with the preponderance of evidence from studies in IBD patients, reduction in systemic inflammation following prednisolone or infliximab treatment was associated with an increase in circulating IGF-I levels [3, 5, 7, 37]. Infliximab, but not prednisolone, increased IGF-II and IGFBP-3. However, in contrast to our expectations, prednisolone, but not infliximab, increased bioactive IGF in serum. As a novel finding, both treatments caused significant reductions in intact and fragmented IGFBP-4 levels. These changes occurred independently of STC2 and PAPP-A, which both remained virtually unaffected by either treatment. Thus, the effect of immunosuppression did not appear to extend beyond the regulation of IGF and IGFBP-4. In conjunction, our study implies that the distinct metabolic effects of prednisolone and infliximab may primarily be regulated at the local tissue level rather than through alterations in the circulating IGF system.

Prednisolone is a glucocorticoid receptor agonist, and its anti-inflammatory actions involve inhibition of potent mediators of inflammation such as cytokines, prostaglandins, and leukotrienes. Infliximab is a chimeric monoclonal antibody that neutralizes the activity of circulating TNF-α, which is primarily produced by activated macrophages in response to inflammation. Infliximab also induces immune cell apoptosis trough reverse signaling via transmembrane TNF-α, thereby reducing the production of other pro-inflammatory cytokines [38–40]. Prednisolone exhibits significant adverse effects, such as insulin resistance and catabolism, whereas metabolic side effects attributable to infliximab are less common. Interestingly, in our study, prednisolone also increased IGF bioactivity, whereas this increment did not follow infliximab administration. Data on the relationship between total and bioactive IGF in response to immune-modulating therapy are equivocal. A recent study in healthy males treated with prednisolone (37.5 mg daily) for 5 days demonstrated augmented total and bioactive IGF-I levels [10]. In contrast, in asthmatic children treated with low dose prednisolone (5 mg daily) for 1 week, IGF bioactivity was diminished despite unaltered total and free IGF-I [12], and IBD patients on 8–12 weeks prednisolone demonstrated increased total IGF-I, whereas free IGF-I was unchanged in patients with Crohn’s disease and increased in ulcerative colitis [41]. Infliximab treatment of patients with Crohn’s disease has been shown to augment total IGF-I, but to suppress free IGF-I levels [7].

It is reasonable to assume that the effects of prednisolone and infliximab on total IGF-I occur primarily as a result of the attenuated inflammation. Furthermore, IGF-I and pro-inflammatory cytokines are disparately regulated, as inflammation leads amino acids away from muscle accretion and growth toward hepatic acute-phase protein synthesis [42]. TNF-α is reported to decrease hepatic GH receptor synthesis and as a result of this, hepatic IGF-I and IGFBP-3 production, and this readily explains why infliximab increases serum IGF-I [3, 7]. In contrast, exposure to prednisolone affects the IGF system at multiple levels, resulting in blunted mRNA expression, hormone release, receptor abundance, and signal transduction [43], yet endocrine IGF-I remains unaltered or even increased [12, 13]. It is contradictory that prednisolone induces an increase in circulating IGF-I and at the same time exerts catabolic effects on muscle and bone by releasing amino acids into the bloodstream to provide substrate supply for hepatic urea synthesis [27]. The dual outcome suggests that the beneficial anti-inflammatory effect of prednisolone on the IGF axis overcomes its adverse catabolic properties. Thus, upon treatment, IGF-I may still remedy the steroid-induced protein wasting and contribute towards whole-body anabolism. Part of the dual effect could also arise from a relieved blockade of GH secretion induced at the hypothalamic-pituitary level, and putatively, serum IGF-I concentration and action may increase in response to prednisolone-induced insulin resistance, whereas it may disrupt IGF-I signaling at the tissue level. Indeed, it was recently demonstrated that prednisolone results in cellular IGF-I resistance by impeding IGF-IR down-stream signaling [10].

There is a strong consensus that IGFBP-4 attenuates IGF bioactivity in most physiological contexts, and thus, prednisolone and infliximab may influence on IGF-I action through the STC2/PAPP-A/IGFBP-4-axis. The idea gains support from studies in murine models, which also have illustrated that the biological function of the axis is not straightforward [36]. Disruption of the PAPP-A gene results in increased abundance of IGFBP-4 that manifests as a 40% growth retardation and an extended lifespan by up to 40% as compared to wild type littermates [20, 44, 45]. Similarly, inactivation of PAPP-A enzymatic activity by transgenic overexpression of STC2 results in a 45% growth deficit [46, 47]. Interestingly, neither of the mouse models show serum IGF-I levels that diverge from those of wild type animals, implying that the axis regulates local IGF bioactivity within the tissues without affecting circulating IGF-I levels [17, 47]. However, in contrast to the expectation, severe growth retardation also accompanies IGFBP-4 deletion, confirming that IGFBP-4 impinge on growth in positive and negative directions and is indeed required for optimal IGF-mediated growth [45, 48, 49].

Our findings following immune-modulating treatment concur with a previous study, in which intact IGFBP-4 and IGFBP-4 fragments were equally suppressed in healthy males treated with prednisolone for 5 days, while PAPP-A remained unaffected. In theory, the decrease in intact IGFBP-4 level could be attributed to an increase in PAPP-A proteolytic activity. However, the proportion of IGFBP-4 fragment as compared to intact IGFBP-4 was of similar magnitude before and after either treatment, and thus, the infliximab induced increment in circulating PAPP-A was not reflected in correspondingly low levels of intact IGFBP-4 and elevated levels of IGFBP-4 fragments. Thus, our data yields no evidence that prednisolone affects circulating PAPP-A proteolytic activity differently than infliximab. Instead, our finding suggests that prednisolone and infliximab affect IGFBP-4 protein transcription or translation either directly or indirectly. The IGFBP-4 reductions may in part be due to compensatory mechanisms, with e.g. IGFBP-4 translation being downregulated in response to the pronounced alterations within the IGF system. Furthermore, it is likely that the diminution in IGFBP-4 may not be directly driven by prednisolone or infliximab, but by the ensuing reduction in inflammation. IGFBP-4 has previously been shown to be upregulated upon TNF-α exposure [50, 51], and it is tenable that increased IGFBP-4 production in response to inflammation affects IGF bioavailability. PAPP-A is also notoriously associated with inflammatory states, and both IL-6 and TNF-α are invariably powerful stimulators [26, 52, 53]. However, despite reduced systemic inflammation, neither patient group experienced a reduction in serum PAPP-A levels upon treatment. The infliximab group presented with increased concentrations. In most patients, anti-TNF-α therapy causes a rebound production of soluble TNF-α to counteracts its effects [38], and thus, it is highly plausible that the slight increase in PAPP-A is driven by increased serum TNF-α concentrations [54, 55].

Interestingly, at baseline, intact IGFBP-4 did not differ from healthy controls, yet IGFBP-4 fragment levels were significantly elevated and as were the ratios. This finding indicates an increased total IGFBP-4 synthesis and subsequently increased fragmentation in untreated IBD patients, causing IGFBP-4 production to appear (erroneously) normal. To fit with this hypothesis, PAPP-A, being the principal if not only IGFBP-4 protease [56], ought to be augmented in patients with IBD, but neither levels of total PAPP-A nor STC2 deviated from healthy controls at baseline. However, in the circulation, other inhibitors than STC2 are present (e.g. eosinophil major basic protein), which renders PAPP-A inactive towards it substrates [56]. Unfortunately, PAPP-A assays do not distinguish between enzymatically active and inactive forms, and we cannot preclude that PAPP-A activity is affected in untreated IBD patients by other inhibitors [14]. Evidently, much needs to be learned about the regulation and role of IGFBP-4 in inflammatory states.

In the present work, a number of shortcomings should be addressed. Firstly, patients were only studied for 1 week, and measurements were only conducted on day 0 and day 7. A longer follow-up period would have allowed for complete remission, and repeated measurements would have provided a more detailed picture of the disease improvement. Secondly, the shifts in protein levels may not only be an effect of treatment, but rather a reflection of overall amelioration in disease activity. Finally, GH was not measured in this cohort, and only IGF-I was assessed as a marker of GH action. However, GH measurements may have provided additional information.

## Conclusion

In patients with IBD, IGFBP-4 and its fragments were reduced by prednisolone and infliximab therapy either directly or indirectly in response to the resultant reduction in inflammation. If so, inflammation may be a critical determinant of IGFBP-4 and, to some extent, PAPP-A activity. Thus, the findings add to the growing body of evidence linking the STC2/PAPP-A/IGFBP-4-axis to inflammatory diseases. However, the clinical and pathophysiological importance of this dysregulation, and whether the system partakes in the onset or progression of disease, remains a conundrum and warrants further studies.

## Data Availability

The dataset used and analyzed during the current study is not publicly available due to the General Data Protection Regulation but is available in fully anonymized form from the corresponding author on reasonable request.

## Abbreviations

BMI: Body mass index
CRP: C-reactive protein
CT: C-terminal
eGFR: Estimated glomerular filtration rate
ESR: Erythrocyte sedimentation rate
GH: Growth hormone
HBI: Harvey–Bradshaw Index
IBD: Inflammatory bowel disease
IGF: Insulin-like growth factor
IGFBP: IGF binding protein
IL-6: Interleukin-6
KIRA: Kinase receptor activation assay
NS: Not significant
NT: N-terminal
PAPP-A: Pregnancy-associated plasma protein-A
SCCAI: Simple Clinical Colitis Activity Index
STC2: Stanniocalcin-2
TNF-α: Tumour necrosis factor-α
